# Community pharmacists’ views and experiences of delivering in-pharmacy medication reviews for people living with severe and persistent mental illness: a qualitative study

**DOI:** 10.1007/s11096-024-01720-2

**Published:** 2024-03-29

**Authors:** Ricki Ng, Sarira El-Den, Jack C. Collins, Sara S. McMillan, Jie Hu, Amanda J. Wheeler, Claire L. O’Reilly

**Affiliations:** 1 The University of Sydney School of Pharmacy, Faculty of Medicine and Health, The University of Sydney, Camperdown, Australia; 2 Centre for Mental Health and Menzies Health Institute Queensland, Griffith University, Brisbane, Australia; 3 School of Pharmacy and Medical Sciences, Griffith Health, Griffith University, Gold Coast, Australia; 4 Faculty of Medical and Health Sciences, University of Auckland, Auckland, New Zealand

**Keywords:** Community pharmacist, Community pharmacy services, Drug utilisation review, Mental health, Primary medical care

## Abstract

**Background:**

People living with severe and persistent mental illness (SPMI) often take multiple medications and are at risk of experiencing medication related problems. Medication review services have the potential to reduce inappropriate use of psychotropic medications and improve adherence. However, there is limited research regarding pharmacists’ perspectives when providing such services.

**Aim:**

To explore community pharmacists’ views and experiences of providing an in-pharmacy medication review (MedsCheck) for people living with SPMI.

**Method:**

Semi-structured interviews were conducted between November 2021 and May 2022 with community pharmacists participating in the comparator group of the *PharMIbridge* Randomised Controlled Trial (RCT), which aimed to improve medication adherence and manage physical health concerns for people living with SPMI. Interviews were recorded, transcribed, and analysed using inductive thematic analysis.

**Results:**

Fifteen semi-structured interviews were conducted with community pharmacists including pharmacy owners, managers and employee pharmacists. Most pharmacist participants who were interviewed (n = 10) were aged under 39 and more than half (n = 8) had 10 or more years of pharmacy experience. Five key themes were identified: 1) Pharmacists’ roles in the management of SPMI in community pharmacy; 2) Mental health education and training; 3) Pharmacy resources; 4) Challenges with interprofessional collaboration and 5) Impact on professional relationships and consumer outcomes.

**Conclusion:**

Pharmacists are motivated to support people living with SPMI. Mental health training, as well as arrangements regarding pharmacy workflow and appropriate remuneration are needed to enable pharmacists to better support people living with SPMI. Referral pathways should be directly accessible by community pharmacists to assist interprofessional collaboration.

**Supplementary Information:**

The online version contains supplementary material available at 10.1007/s11096-024-01720-2.

## Impact statements

- Pharmacists were motivated to support people living with SPMI through in-pharmacy medication review services, if appropriate training in mental health for pharmacists and pharmacy staff is provided.
- Policy makers should consider alternative remuneration pathways to ensure that pharmacists are appropriately compensated for their work and that there is sufficient time and staffing available to ensure the feasibility and sustainability of medication review services for people living with SPMI.
- Improving interprofessional collaboration and enhancing pharmacy workflow are the proposed mechanisms to facilitate community pharmacists’ roles in supporting people living with SPMI, which provide a basis for better understanding the factors contributing to the implementation of community pharmacy mental health services.

## Introduction

Internationally, many countries have implemented pharmacist-led medication review programs to improve health outcomes [1, 2]. Medication reviews involve “a structured evaluation of a patient’s medicines with the aim of optimising medicines use and improving health outcomes” [3]. Medication review is a key element of medicines use optimisation that has been recommended by national and international policy documents and guidelines for people taking multiple medications [4, 5]. People living with severe and persistent mental illness (SPMI), of which include conditions such as bipolar disorder and schizophrenia [6], commonly experience psychotropic polypharmacy and regular medication reviews can ensure that medications prescribed are safe and effective [7]. Partly due to comorbid health conditions such as cardiometabolic diseases and cancer [8, 9], people living with SPMI die up to 10–25 years earlier than the general population [10]. Poor physical health outcomes experienced by this population have been attributed to a combination of different factors such as the adverse effects of psychotropic medications and having limited access to healthcare resources [11], highlighting the opportunity for pharmacists to provide further support.

Community pharmacists across the world offer medication review services to consumers^1^ to ensure the safe and effective use of medicines, identify potential drug interactions, and improve consumer understanding [4, 12]. For instance, in Australia, the MedsCheck service is a government funded medication review service that can be provided by all community pharmacists. It aims to address consumers’ medication-related concerns and improve their understanding of their medicines, whereas the Home Medicines Review is a more comprehensive medication review that is provided by a consultant pharmacist upon referral from a general practitioner (GP). Alternatively, in New Zealand, the Medicines Use Review is provided by accredited pharmacists in a variety of settings including the person’s home, community or hospital pharmacy [13]. Similarly, the Medicines Use Review in the United Kingdom is a structured medication review during a face-to-face consultation between a pharmacist and consumer [14]. However, all of these services differ in consumer eligibility, intervention content and modes of delivery across countries [2], and currently none of these are focused on mental health conditions.

There is a growing interest in the role of community pharmacists in improving the medication management of people living with mental illness [15, 16]. For example, pharmacists have demonstrated their capacity to conduct mental health screening to identify consumers at risk of depression [17], and to educate consumers to improve adherence to antidepressants [18]. In 2020–2021, the Bridging the Gap between Physical and Mental Illness in Community Pharmacy (*PharMIbridge*) Randomised Controlled Trial (RCT) was conducted in Australia [19], which aimed to investigate the effectiveness of a person-centred, goal-oriented pharmacist-led support service for people living with SPMI, compared to a standard in-pharmacy medication review service (MedsCheck). Previous research has explored pharmacists’ experiences in delivering medication reviews [20, 21], and specifically MedsChecks for people living with diabetes [22], however pharmacists’ views and experiences with providing MedsChecks for people living with SPMI have not been explored.

### Aim

To explore *PharMIbridge* community pharmacists’ views and experiences of providing MedsChecks for people living with SPMI.

### Ethics approval

Ethical approval was granted by Griffith University Human Research Ethics Committee (Project number: HREC/2019/473).

## Method

As part of the *PharMIbridge* RCT [19], this study reported on the views and experiences of comparator group (CG) pharmacists who delivered the MedsCheck service for people living with SPMI. Consumer participant eligibility for the *PharMIbridge* RCT is reported elsewhere [19]. Recruited pharmacies were randomised to either the Intervention Group (IG) or CG. Further information regarding the IG, including the *PharMIbridge* intervention and *PharMIbridge* training, has been described previously [19, 23–25]. Twenty-six pharmacies were randomised to the CG with 68 pharmacists attending the CG training, which included training on participant recruitment and study processes for the RCT, including consumer eligibility, informed consent and service documentation [25] and Mental Health First Aid (MHFA) [26]. It was a requirement for CG pharmacists to attend MHFA training to ensure that they were able to support people who might experience any psychological distress or mental health crises during the RCT [26]. The Standards for Reporting Qualitative Research (SRQR) were used for the reporting of this study [27].

### Pharmacist participants

Pharmacists were recruited through community pharmacies as part of the *PharMIbridge* RCT [19]. After RCT completion, all CG pharmacists were invited to participate in an interview. Pharmacists were provided with the participant information statement and consent form prior to the interview via email. Pharmacists were given an AUD30 gift voucher after the interview.

### Data collection

Based on previously published studies [28, 29], a semi-structured interview guide was developed by the research team to explore pharmacists’ experiences of delivering the MedsCheck service as part of the *PharMIbridge* RCT (see electronic supplementary material 1). Semi-structured interviews were conducted via telephone by one researcher (RN) between November 2021 and May 2022. Interview audio-recordings were de-identified and transcribed verbatim for analysis. Transcripts were quality checked by the interviewer for accuracy. Demographic data including participant’s age, gender, role(s) in the pharmacy and years of pharmacy experience were also collected.

### Data analysis

Data analysis occurred after completion of all interviews. To explore pharmacists’ experiences with providing MedsChecks to consumer participants living with SPMI, qualitative thematic analysis was conducted using a six-phase approach as outlined by Braun and Clarke [30]. One researcher (RN) listened to the recordings and read the transcripts to familiarise themselves with the data. RN is a practising community pharmacist, trained in qualitative research methods and had limited involvement with participating pharmacists in the *PharMIbridge* RCT. A second researcher (COR, a pharmacist, pharmacy and mental health researcher) independently coded a random 25% sample of interview transcripts. Personal reflexivity was taken into consideration when analysing and interpretating the findings of the research. RN and COR initially coded the data inductively and independently from the transcripts in Microsoft Word ® where each key feature was highlighted, and the relevant code was documented. Through discussions, codes were then refined by two researchers (RN and COR) and a coding framework was developed. The identified emerging patterns from the codes were then used to create themes and sub-themes. Through examining the coded data and having iterative discussions with other researchers in the team (SE, JC and COR), final themes were developed.

## Results

### Participant characteristics

Fifteen community pharmacists were interviewed. At the time of the interviews, pharmacists were aged between 25 and 53 years (mean 36.9; SD 8.77). Eight pharmacists (53.3%) had 10 or more years of pharmacy experience (Table 1). Table 2 provides information about each participant.

**Table 1:** Interview participant characteristics (n = 15)

Characteristics		n
Gender	Male	7
	Female	8
Age (years)	< 30	5
	30–39	5
	40–49	3
	50–59	2
Role	Pharmacist	6
	Pharmacist in charge/pharmacy manager	4
	Pharmacist and pharmacy owner	5
Pharmacy experience (years)	1–9	7
	10–19	5
	> 20	3

**Table 2:** Demographic characteristics of participants

Participant	Gender	Role
P1	Male	Pharmacy manager
P2	Female	Pharmacist
P3	Female	Pharmacist
P4	Male	Pharmacist
P5	Male	Pharmacist in charge
P6	Female	Pharmacy owner
P7	Male	Pharmacist manager
P8	Female	Pharmacist owner
P9	Female	Pharmacist owner
P10	Female	Pharmacy owner
P11	Female	Pharmacist
P12	Male	Pharmacy owner
P13	Female	Pharmacist
P14	Male	Pharmacist in charge
P15	Male	Pharmacist

Five themes were generated including:

1. Pharmacists’ roles in the management of SPMI in community pharmacy
2. Mental health education and training
3. Pharmacy resources
4. Challenges with interprofessional collaboration
5. Impact on professional relationships and consumer outcomes

#### Pharmacists’ roles in the management of SPMI in community pharmacy

Most pharmacists stated that they were motivated to be supporting and working with people living with SPMI. Pharmacists welcomed the idea of having a mental health specific MedsCheck service, as it would allow for a comprehensive assessment of medication-related issues in the context of mental health.

“I would say that a mental health check MedsCheck is far more beneficial than just your regular MedsCheck, because there’s so many more important things that you can pick up with mental health than someone who’s just got hypertension.” (P6)

However, rather than a regular medication review, pharmacists mentioned how they could serve as an alternative point of contact for consumers to discuss mental health issues and recognised the need for a pharmacy-based service that would target both the clinical and wellbeing needs of consumers living with SPMI. They also reported on how community pharmacies were more accessible, overcoming access and time constraints that doctors may have.

“Going through the medications offers little benefit if it’s done in…a black and white way…the majority of benefit was through actually having the person sit down and go through... whatever issues that they’re feeling [or]…Having the ability to get things off their chest that they might not actually have the ability to do through prescribers either if it’s time constrained or whatever reason.” (P14)

Pharmacists reported how pharmacies became the “*safe spot*” (P5) to discuss their mental health concerns. It enabled pharmacists to adopt a proactive approach, focusing on prevention rather than treatment.

“The flow on effect to their health is obvious…If something’s going wrong, they’ll be more inclined to come forward early rather than later, if they don’t get the feeling that they’re being judged, or that it’s a place where they’re safe to come and express that things are going wrong.” (P10)

#### Mental health education and training

Most pharmacists commented that MHFA training helped them to be more confident in assisting and supporting consumers living with SPMI. Pharmacists highlighted it is critical to have mental health crisis skills training such as MHFA when supporting consumers living with SPMI through a MedsCheck.

“To have that Mental Health First Aid accreditation, I was able to deal with [mental health situations] rather than just be an ordinary pharmacist who’s drawing information from our studies at university and everyday experiences. I think having that qualification behind us helped me with confidence and wanting to be a bit more proactive about it.” (P7)

Several pharmacists mentioned how MHFA training corrected their misconceptions around suicide and self-harm. After MHFA training, pharmacists reported being able to utilise the skills learnt around suicide assessment and apply them to their everyday pharmacy practice.

“I’m not afraid to ask the [suicide] question if they’re considering self-harm, whereas perhaps in the past I would balk at that question, but now I can be quite upfront about asking that.” (P10)

Pharmacists reported that real life experiences helped consolidate the knowledge learnt in MHFA training and described how it further improved their confidence in assisting with mental health situations.

“Experience is the key in terms of being able to successfully manage or communicate with people who may be experiencing acute mental health presentations or just general mental health clientele. [MHFA] definitely gives [me] a good foundation to be able to start to feel comfortable dealing with those situations. But at the end of the day, it was actual experience in dealing with real matters in real life that actually made me confident.” (P14)

Despite MHFA training, a few pharmacists expressed desire for further mental health training. They reflected on the need go beyond basic pharmacotherapy and delve into the practical applications and use of antipsychotic medications through specialised training to be able to confidently provide a MedsCheck to people living with SPMI.

“Do a special training on antipsychotics…in depth…We actually know the surface of it because we’ve got the basic pharmacology, but what is really being used [in] practice?” (P8)

#### Pharmacy resources

Most pharmacists faced challenges with balancing their time between providing MedsChecks as part of the *PharMIbridge* RCT and maintaining normal pharmacy operations. All pharmacists mentioned that MedsChecks for people living with SPMI were more time-consuming.

“You need a bit more time…With these ones, you [need to] sit down with the patient and they might offload a bit more on how they’re feeling.” (P12)

An additional challenge in terms of pharmacy resources was the lack of sufficient pharmacists on duty. Due to COVID-19 restrictions, pharmacies had to “split teams and [were] short staffed.” (P2). Moreover, pharmacists described the challenges of providing the MedsCheck service if they were the solo pharmacist in the pharmacy.

“The main thing for us [was]…staffing…We don’t have enough pharmacist…So it’s only the time when there are two pharmacists available that we can do [MedsChecks].*”* (P2)

Pharmacists believed that having adequate staff on duty was necessary to support pharmacies to have one pharmacist focus on delivering professional services (e.g., MedsChecks) while others manage pharmacy operational functions. Pharmacists also mentioned the need for having appropriate remuneration to compensate for the need to hire additional staff and time spent for delivering MedsChecks to consumers living with SPMI.

“You have to have the staff to do it or… someone who you’re happy to pay their hourly rate to sit down for half an hour with someone. So, there has to be a financial component.” (P6)

#### Challenges with interprofessional collaboration

Pharmacists recognised the importance of collaborating with local GPs and mental healthcare teams. Whilst pharmacists attempted to communicate with other healthcare providers, pharmacists reported that the communication process was not smooth and often delayed.

“[It’s] just abysmal for trying to get onto any specialists at the hospital, or elsewhere. It’s a joke [to] try and call and get a response three days later. So, [we] tend to be pretty good at problem solving without them.” (P4)

Many pharmacists argued that the current mental healthcare system does not encourage collaboration or communication among healthcare professionals, and that the nature of the interactions between pharmacists and other healthcare professionals does not happen beyond the scope of an immediate need to solve a problem.

“When it comes to psychologists and psychiatrists, there really isn’t much of a collaboration [with pharmacists] unless there’s a query regarding medication for a patient or we need to speak to them.” (P7)

In Australia, pharmacists typically refer to the GP as the coordinator of care who then makes referrals to other health services such as psychologists. Pharmacists may facilitate the process of referring consumers to another healthcare professional, but only referrals from a GP qualify for subsidy, which may be a barrier for pharmacists to be actively involved in interprofessional collaboration. Pharmacists believed that having a more direct referral approach would give them greater capacity to address mental health and wellbeing problems and alleviate burden on other healthcare professionals.

“Our system is broken and needs to be re-engineered. [Pharmacists] should be able to refer to psychologists that can access funding. But unfortunately, that isn’t the case [currently]…the GP has to provide a…Team Care Arrangement…which then enables the person to [be referred]…to the psychologist…That is so out of touch with reality and an overburdened healthcare system…We need to have…programs in place to help ease some of that burden on…other allied health professionals.” (P12)

In the absence of specific referral pathways, pharmacists described how they were uncertain about the care pathways that were available to refer consumers to or supports and resources accessible to them during a mental health crisis situation.

“I don’t know who to call. I had to Google who to call…There’s really no triple-zero [emergency phone number] in mental health crisis except the CATT [Crisis Assessment and Treatment Teams] I suppose.” (P8)

Some pharmacists considered that the lack of understanding of pharmacists’ skills and expertise in mental healthcare among health professionals also seemed to impede the collaborative process. A few pharmacists faced resistance when they made efforts to engage with other members of the mental health team especially if they had identified someone in crisis who needed further support.

“[The mental health crisis team] are not really open minded in sharing their roles…[The team]…said… “What training have you done to be able to handle this crisis?” And I…was taken back with that. I was like, “I’ll help you as a team member here…we did Mental Health First Aid”, and [they] first chuckled …almost…patronising, [said] “You’ll need more than that”…. I don’t know if they would actually take me more seriously if I were a doctor or a nurse…But…they definitely didn’t expect a call from the pharmacy.” (P8)

#### Impact on professional relationships and consumer outcomes

Pharmacists acknowledged the importance of building relationships with people living with SPMI and mentioned that MedsChecks were helpful to engage with consumers and strengthen their relationships. Having rapport and trust was seen as essential in the relationship, which helped consumers feel comfortable to open up and discuss their health concerns with the pharmacist.

“The biggest positive take-home has been that rapport with the customers…[They] have no hesitations coming up to either myself or the other pharmacist on-duty to talk about even small elements of their medical care…that aren’t about their mental health, or issues that are just jovial in nature.” (P5)

This was echoed by another pharmacist who described that building rapport with consumers could potentially lead to improvements in medication adherence.

“They open up a little bit more and…talk more openly…[The] relationship that we built with the patient…also [helped] with…compliance…when the relationship is improved.” (P1)

Pharmacists provided education to consumers during the MedsCheck service to correct their misconceptions and improve their medication-related understanding, stating *“even though they thought they knew what their medication was for…a lot of them actually didn’t really understand exactly what it did or how they worked.”* (P6). By addressing health literacy, pharmacists reported how it could empower consumers by giving them the knowledge they needed to self-manage their medications and health conditions.

“They have a greater medication knowledge now because of it and understand why they’re taking a lot of things. Whereas before they were just taking them because of doctor wrote a script.” (P11)

## Discussion

### Statement of key findings

Pharmacists were motivated and articulated the support that they could provide to consumers living with SPMI. While mental health training and education enabled and empowered pharmacists to deliver MedsChecks for people living with SPMI, several barriers such as time constraints and staffing issues in the pharmacy and challenges involving other healthcare professionals can affect pharmacists’ ability to support this population. Nonetheless, pharmacists described the benefits experienced by consumers following a MedsCheck and supported the idea of having mental health specific medication review services, rather than a regular medication review.

### Strengths and weaknesses

This is the first study that provides insights into the perspectives and experiences of community pharmacists providing the MedsCheck service to people living with SPMI. Community pharmacies were recruited from different states and practice locations, which provided a variety of insights into the experiences of pharmacists from several geographical locations. However, it should be noted that only pharmacists participating in the *PharMIbridge* RCT were interviewed, which may limit transferability [31]. The findings were in the context of the RCT, and as such, it remains largely unknown how pharmacists’ perspectives may differ outside the controlled trial environment.

The interviewer’s experience as a community pharmacist facilitated an immediate connection with the pharmacists interviewed. However, it is recognised that the personal experience of the interviewer could introduce an element of bias. Therefore, a second researcher coded 25% of the transcripts and subsequent themes developed from the data to ensure data analysis was comprehensive [32]. Furthermore, participation in the interviews was based on the availability of the pharmacists, which may lead to sampling and selection bias. However, the sample size appeared to be adequate as no additional themes emerged from the data towards the end of the analysis.

### Interpretation

The findings showed the potential value of pharmacists’ involvement in mental healthcare. Pharmacists’ scope of practice is expanding and evolving rapidly in response to the overburdened healthcare system in Australia [33, 34]. Evidence regarding the involvement of community pharmacists in mental healthcare is growing [35, 36], and the current study adds to the existing evidence that pharmacists can support people living with SPMI through medication review services. Through MedsChecks, pharmacists were able to build rapport and trust with consumers, making community pharmacies potentially a safe space for people living with SPMI to discuss their mental health and medication concerns. Pharmacists also reflected on the potential role they could play in mental healthcare in improving consumer health outcomes and the importance of services targeting the holistic needs of people living with SPMI.

The findings indicated that the issues relating to lack of pharmacy resources affected pharmacists in their ability to provide care to people living with SPMI. Although MedsCheck services are routinely provided in Australian community pharmacies, pharmacists reported a MedsCheck for people living with SPMI required more time and effort due to the complexity of SPMI conditions. This is in line with the evidence regarding the delivery of mental healthcare in general practice, which identified that mental healthcare is more complex and often requires more time than a standard consultation [37]. The report exploring GPs providing mental health consultations suggested the need to appropriately remunerate GPs for the additional time spent to coordinate care and follow-up with mental health consumers. While the current research on pharmacy consultation times remains limited in Australia [38], there is a need to appropriately remunerate pharmacists for the additional time spent providing MedsChecks for mental health consumers. Once pharmacists receive adequate remuneration to support people living with mental illness, a more robust assessment of pharmacy workflow is needed to reallocate resources, which may allow pharmacists to devote more time for MedsChecks for people living with SPMI.

In addition to in-pharmacy challenges, as other researchers have found [39, 40], pharmacists described the disconnect with other healthcare professionals and expressed the need for more collaboration and communication. Unfortunately, community pharmacists are often overlooked in the context of providing multidisciplinary team based collaborative care to people living with mental illness [41]. Previous studies have demonstrated that fragmentation of services within healthcare systems can make collaboration challenging, leading to less integrated care [42, 43]. A stepwise approach of raising awareness and promoting interprofessional education among healthcare professionals is a potential solution for improving collaboration [44]. While education has the potential to positively impact the future of interprofessional collaboration, research has suggested the need to establish a system for different health disciplines to work together for more coordinated care [45]. This may be established by integrating pharmacists to mental healthcare teams or having better referral pathways in place, which would allow pharmacists to refer consumers or be directed for medication related issues or consultations. Nonetheless, more research is needed in this area to optimise the linkage between other healthcare professionals and the individual services offered by pharmacists.

While lack of knowledge has been identified as a barrier in the provision of pharmacy services to people living with mental illness [46], pharmacists in the study who had received MHFA training reported that they were able to apply the acquired skills in real-life contexts when delivering MedsCheck services. MHFA training is one approach to upskill pharmacists in mental health and should be considered during the implementation or provision of pharmacy services for people living with SPMI [47–50]. There have been calls for all community pharmacists to complete MHFA training in order to assist consumers to get the right support when they need it [48, 49]. Research has demonstrated that pharmacists are regularly exposed to people in crisis [47, 51], which highlighted the importance of mental health and/or crisis training. On a similar way to physical first aid training for pharmacists in Australia [52], MHFA training should be encouraged for all practising pharmacists to increase their level of mental health competence.

### Further research

Building from the findings from this study, future research could investigate ways to better support pharmacists in the provision of pharmacy services for people living with SPMI. Having enough time and staff on duty have been identified to influence the provision of the MedsCheck service for people living with SPMI. Considerations regarding pharmacy workflow and remuneration are needed to appropriately compensate pharmacists for their work and ensure that such services are sustainable. Future research should explore other healthcare professionals’ (e.g., GPs) perspectives of the MedsCheck service to ensure that the perspectives of all healthcare professionals are captured and allow policy makers to make informed decisions that benefit the community.

## Conclusion

This study identified an opportunity and a need to better utilise community pharmacists in supporting people living with SPMI. With appropriate training, the MedsCheck service presented an opportunity for pharmacists to engage in wider discussions of consumers’ mental health. The study also highlighted the challenges such as issues related to time management and interprofessional collaboration as more pharmacies move towards providing and implementing an increasing range of professional pharmacy care services.

### Supplementary Information

Below is the link to the electronic supplementary material.

Supplementary file1 (DOCX 25 KB)

## Footnotes

1 The terms ‘consumer’, ‘patient’, ‘client’ and ‘service user’ may be used depending on the setting or context. The term ‘consumer’ is used by the authors to describe a person visiting a pharmacy to purchase products, obtain medications, or seek advice.
