# Supplementary material for: Community pharmacists’ views and experiences of delivering in-pharmacy medication reviews for people living with severe and persistent mental illness: a qualitative study
Source: Int J Clin Pharm. 2024 Mar 29;46(4):862–71. doi: 10.1007/s11096-024-01720-2 (PMC11286626; doi:10.1007/s11096-024-01720-2)
Supplement: Supplementary file 1 — Supplementary file1 (DOCX 25 KB) [file 11096_2024_1720_MOESM1_ESM.docx]

**Pharmacist evaluation of the *PharMIbridge* Medscheck Service**

**Interview Guide - CG**

**Instructions to Interviewer:**

This interview will be conducted with Control Group (CG) pharmacists that participated in the *PharMIbridge* RCT. The interview will be undertaken at RCT completion.

Interviews will explore pharmacists’ acceptability of the *PharMIbridge* MedsCheck service and experiences of being involved in the RCT.

Interviews will be conducted face-to-face, by telephone or through an online meeting platform (e.g. Zoom or Microsoft Teams), at a mutually convenient time for pharmacy and research staff.

Please note that this guide serves only to prompt the interviewer; it is not intended to be prescriptive. Hence, it is likely that the order of questions will change and that some questions will be omitted.

**Introduction**

Introduce yourself.

*Thank you for your time. The purpose of this interview is to ask you about your involvement with delivering the Medscheck service as part of the PharMIbridge RCT, the associated training and your experiences working with people living with severe and persistent mental illness (SPMI). We also want to hear about any positive outcomes and challenges you faced as this will help us with future service implementation.*

*We value your input and your honest contribution. Anything you tell us will be treated with the strictest confidence and your identity will be protected. You are free to skip questions that you prefer not to answer or finish the interview at any time.*

***The interview should take about 30 minutes.***

*Have you read the information sheet?*

- NB. If participant has not read the sheet, discuss the information sheet

*Do you have any questions about the interview?*

- Answer all questions

*Are you happy for me to commence recording of the consent process?*

- If not, will need written consent.

Read out consent form. If the participant does not wish the interview to be recorded, recording needs to stop and handwritten notes made.

**START INTERVIEW**

**INTERVIEW QUESTIONS**

**I’d like to begin by asking some questions about the *PharMIbridge* Comparator Group training that you received.**

1. *How did the 1-day PharMIbridge delivery training sessions support you to participate in the trial and provide a MedsCheck to consumers living with SPMI?*

- ***How did the PharMIbridge MedsCheck delivery training* (remind participants that it is the session where they learnt about recruitment) *help you in obtaining consent, recruiting, collecting baseline information, and following up consumer participants at 6 months?***
- ***Did you feel the training helped you establish your pharmacy as a mental health friendly pharmacy?***
- ***If so, how?***
- ***Do you have any suggested changes or improvements for the training?***
- ***Are there any other topics or activities that you think would have been beneficial to include that were not covered in the training?***

1. *Did you complete Mental Health First Aid (MHFA) training as part of the PharMIbridge training?*

If yes, ask some of the following questions: (if no, still ask the following questions and ask the year of completing MHFA training)

- *How did the MHFA training equip you to support people experiencing mental health crises (e.g. suicidal thoughts, acute psychotic episodes) or people experiencing high distress?*
- *How did MHFA training help you support people living with SPMI?*
- *Were there any situations where you applied MHFA principles that you would like to share?*
- *How did MHFA training affect your practice in the pharmacy, more generally?*

**I’d now like to ask about how *PharMIbridge* may have changed the way that you work with consumers living with SPMI**

1. *How did the PharMIbridge MedsCheck service work in the pharmacy to support people living with SMPI?*
   - *Describe how your pharmacy implemented the PharMIbridge MedsCheck service?* (Prompt: *what worked, what did not work, what would you change?)*
   - *Were there any challenges?* (Prompt: *Research implementation challenges? Pharmacy environment challenges? Challenges with recruitment and consent of consumers? Challenges with other HCPs?)*
   - *If you had the opportunity to start again, would you do anything differently? If yes, please elaborate.*
2. *Can you please describe the* ***key impacts*** *of the PharMIbridge MedsCheck service for consumers?*
   - *What were the outcomes for consumers?* (Prompt: *medication adherence, physical health, mental health and other consumer outcomes*
3. How easy or difficult did you find the process of consumer follow-up at 6 months? Why?

- ***How did you overcome any difficulties? What helped?***

**I’d like to ask you about delivering the *PharMIbridge MedsCheck* service.**

1. ***Can you tell me what resources you used during the RCT?***
   - ***How useful were the PharMIbridge resources (e.g. those available on sharepoint/ service manual) / Research Team site visits or phone calls?***
   - ***Were there any resources that you thought were missing?***
2. ***How feasible do you think it would be to implement a mental health specific MedsCheck service (like the PharMIbridge MedsCheck) more broadly (as a program in more pharmacies, locations?)***
   - *Do you think that the existing MedsCheck service is sufficient to support people living with SPMI?*
   - *If not, do you think that there should be a specific MedsCheck service specific for people living with SPMI?*
     - *Do you have any recommendations for how this might happen?*

**Now you have been involved in the *PharMIbridge MedsCheck*, I would like to ask you about how it has impacted on your pharmacy practice in general.**

1. *How did the PharMIbridge MedsCheck service impact your delivery of the existing MedsCheck service?*

- *Is there any aspect/s of the PharMIbridge MedsCheck service that you will continue to integrate into your practice in the future?*

1. *How did the PharMIbridge MedsCheck service impact your role or practice as a pharmacist?*

- *Any benefits or changes to your role as a pharmacist?*

Prompts:

- - Knowledge (e.g. medication-related, mental health-related, about consumers)
  - Confidence (e.g. working with mental health consumers, contacting other healthcare professionals (HCPs))
  - Comfort (e.g. working with mental health consumers, contacting other HCPs)
  - Relationships/networks (e.g. with consumers / other HCPs / other staff)
  - Operational / procedures

1. *How did the PharMIbridge MedsCheck service affect the pharmacy?*
   - *How did the* *PharMIbridge service benefit the pharmacy? E.g. professional image / profile of your pharmacy*
   - *Impact on other staff within the pharmacy?*
   - *Impact on relationships with doctors/psychologists/psychiatrists/other HCPs?*
2. *As a pharmacist what was it like to participate in a RCT/research project?*

- *What was your expectations of the research process?*
- *What could have been done differently?*
- *Would you recommend other pharmacists participate in research?*

1. Is there anything else you would like to share about your experience in the *PharMIbridge* RCT?

**DEMOGRAPHIC INFORMATION** *– please complete this section for all interviewees*

What best describes your gender?

Age?

Role/title?

Professional qualifications?

Years of pharmacy experience?

Time worked in current pharmacy?

**Thank you for taking the time to talk with us today. We very much appreciate your contribution to the *PharMIbridge* RCT.**

- **Stop recording**
- **Would you like a copy of the transcript?**
- **What is the best email address to send your gift voucher to?**
